# Endo-xylanases as tools for production of substituted xylooligosaccharides with prebiotic properties

**DOI:** 10.1007/s00253-018-9343-4

**Published:** 2018-09-08

**Authors:** Eva Nordberg Karlsson, Eva Schmitz, Javier A. Linares-Pastén, Patrick Adlercreutz

**Affiliations:** Division of Biotechnology, Lund University, P.O.Box 124, 221 00 Lund, Sweden

**Keywords:** Xylanase, Oligosaccharide, Prebiotics, Arabinose, Uronic acids

## Abstract

Xylan has a main chain consisting of β-1,4-linked xylose residues with diverse substituents. Endoxylanases cleave the xylan chain at cleavage sites determined by the substitution pattern and thus give different oligosaccharide product patterns. Most known endoxylanases belong to glycoside hydrolase (GH) families 10 and 11. These enzymes work well on unsubstituted xylan but accept substituents in certain subsites. The GH11 enzymes are more restricted by substituents, but on the other hand, they are normally more active than the GH10 enzymes on insoluble substrates, because of their smaller size. GH5 endoxylanases accept arabinose substituents in several subsites and require it in the − 1 subsite. This specificity makes the GH5 endoxylanases very useful for degradation of highly arabinose-substituted xylans and for the selective production of arabinoxylooligosaccharides, without formation of unsubstituted xylooligosaccharides. The GH30 endoxylanases have a related type of specificity in that they require a uronic acid substituent in the − 2 subsite, which makes them very useful for the production of uronic acid substituted oligosaccharides. The ability of dietary xylooligosaccharides to function as prebiotics in humans is governed by their substitution patterns. Endoxylanases are thus excellent tools to tailor prebiotic oligosaccharides to stimulate various types of intestinal bacteria and to cause fermentation in different parts of the gastrointestinal tract. Continuously increasing knowledge on the function of the gut microbiota and discoveries of novel endoxylanases increase the possibilities to achieve health-promoting effects.

## Introduction

Xylan is the most abundant hemicellulose on Earth, accounting for approximately one third of the renewable organic carbon, and its hydrolysis is performed by different types of xylanases (Collins et al. 2005). These enzymes are produced by a range of different microorganisms, and most of the main-chain acting endoxylanases known to date have evolved from two main scaffolds: the TIM-barrel (α/β)_8_ (found in three different glycoside hydrolase (GH) families of xylanases: GH5, GH10, and GH30) and the β-jelly roll (GH11), which are all retaining enzymes with a double-displacement mechanism. Dependent on the enzyme family, xylans with different substituents and with different degrees of substitution can be hydrolyzed.

**Fig. 1:**
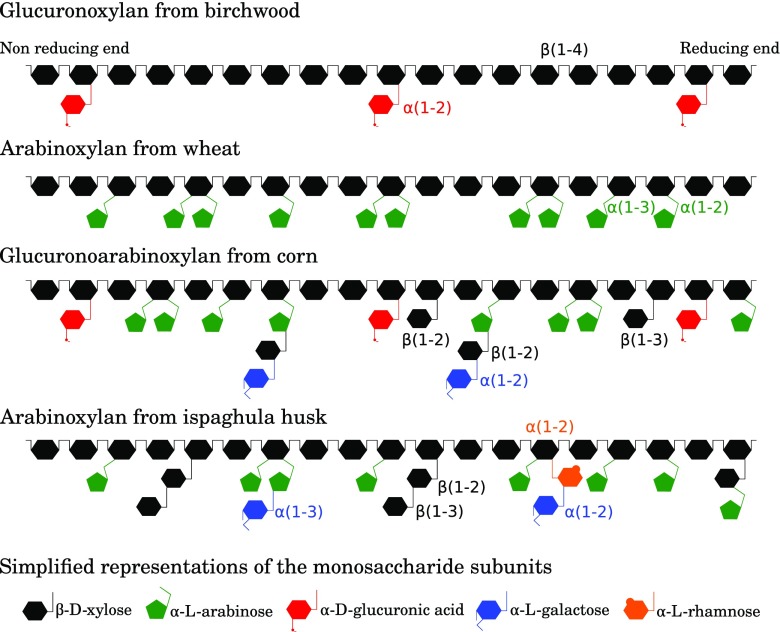
Overview on the structures of the main xylans. Acetyl, methyl, and feruloyl groups are not shown in the structures. Some xyloses of the main chain can be acetylated, 4-O-methylations are common in the glucuronic acid, and some arabinoses can be feruloylated (Based on Edwards et al. 2003; Rogowski et al. 2015)

Xylan is built from a backbone of β-1,4-linked xylose units with diverse substituent decorations that are dependent on both the source and the tissue in the plant where they occur (Stephen 1983; Saha 2003). The substituents include different side chain carbohydrates (Fig. 1) and acids, including uronic acids (Fig. 1), phenolic acids and acetyl groups (Stephen 1983; Biely et al. 2016). Xylans are components of many types of complex biomass, and in most cases, they are underutilized industrially. There is thus considerable potential in obtaining xylans from industrial side streams and upgrade them to valuable products, like prebiotics. Examples of suitable side streams include those from processing of cereals in the food and bio-energy industries.

There has been a tremendous increase in our knowledge on the complete enzymatic degradation of different types of xylans during the last 20 years (Biely et al. 2016). Together with the increased knowledge on how to degrade these polymers, there has however also been an increased interest to preserve some of the features embedded in the polymers, and hence, limited degradation to keep some of the substituents in the polymer is being more and more desirable. An area of interest in this field is enzymatic production of prebiotic oligosaccharides (Linares-Pastén et al. 2018). For this purpose, selective use of main chain-acting endoxylanases is desirable. Produced oligosaccharides can be of interest for ingredient use and will after ingestion be further degraded by the human gut microbiota, resulting in positive health effects (Falck et al. 2013; Berger et al. 2014).

In this mini-review, recent development in the use of endoxylanases to produce substituted xylooligosaccharides (XOSs) will be described. The focus will be on production of oligosaccharides which can be used to promote the growth of beneficial microorganisms in the gut microbiota. The oligosaccharides should thus not be degraded by human digestive enzymes, but fermented in the gastrointestinal tract. It has been shown that the human genome does not contain genes for xylanases, xylosidases, or arabinofuranosidases and experimental studies have confirmed that XOSs and arabinoxylooligosaccharides (AXOSs) are not degraded by human saliva, artificial gastric juice, pancreatin, or intestinal mucosa homogenate (Broekaert et al. 2011). On the other hand, many microorganisms are able to grow on unsubstituted XOS. More selective stimulation of smaller groups of microorganisms can be achieved by the use of substituted XOS. Important progress has recently been made concerning XOS substituted with arabinose or uronic acid substituents and production of those using endo-xylanases will be the main focus of this mini-review.

## GH10 and GH11 endoxylanases

Current research on enzymatic production of various (A)XOS mainly involves the use of bacterial and fungal enzymes from the glycoside hydrolase families 10 (GH10) and 11 (GH11). These families consist predominantly of endo-1,4-β-xylanases (EC 3.2.1.8), which hydrolyze the β-1,4-linked bonds in the backbone of xylan polymers. As the hydrolysis occurs via the double displacement mechanism, they are furthermore classified as retaining enzymes (Linares-Pastén et al. 2018).

**Fig. 2:**
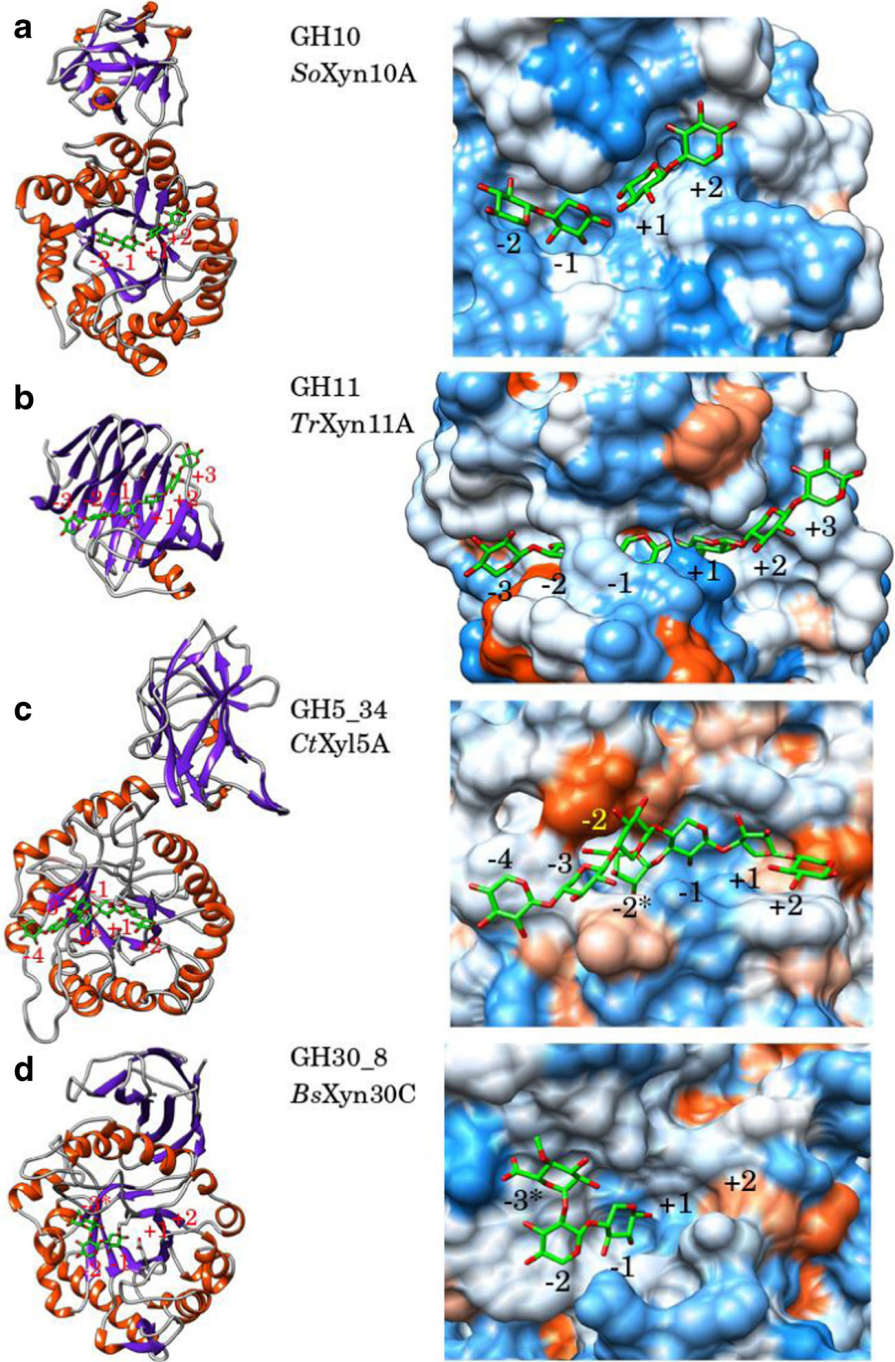
Co-crystallographic structures (enzyme/ligand) of representative xylanases. **a** GH10, *So*XynA10/β-d-Xylp-(1-4)-β-d-Xylp (PDB: 1V6U). **b** GH11, *Tr*Xyn11A/β-d-Xylp-(1-4)-β-D-Xylp-(1-4)-β-d-Xylp-(1-4)-β-d-Xylp-(1-4)-β-d-Xylp-(1–4)-β-d-Xylp (PDB: 4HK8). **c** GH5_34, *Ct*Xyl5A/Xylp-β-1,4-Xylp-β-1,4-Xylp-[α-1,3-Araf]-β-1,4-Xylp-β-1,4-Xylp-β-1,4-Xylp (the aglycone moiety of the ligand was modeled (Falck et al. 2018) based on the co-crystallized structure PDB: 5LA2). **d** GH30_8, *Bs*Xyn30C/α-d-GlcpA4Me-(1-2)-β-D-Xylp-(1-4)-β-D-Xylp (PDB: 3KL5). The hydrophobicity surfaces of the active sites are represented according to the Kyte-Doolittle scale (Kyte and Doolittle 1982), from dodger blue for the most hydrophilic, to white, to orange red for the most hydrophobic. The pictures were made using the software UCSF Chimera v 1.11.2 (Pettersen et al. 2004)

Xylanases belonging to the GH10 family that exhibit the TIM barrel (α/β)_8_ fold typical for clan A enzymes (Fig. 2a). Their active sites consist of well-conserved glycone subsites with strong binding affinity to the substrates and less conserved aglycone subsites with weaker binding affinities. Glycone subsites − 2 to − 1 and − 3 to − 1 strongly bind to xylobiose and xylotriose, respectively. All substrates exceeding these sizes are hydrolyzed at the non-reducing end after three xylose monomers (Biely et al. 1997; Schmidt et al. 1999). Two consecutive unsubstituted xylose monomers are necessary for the GH10 xylanases to cleave the xylan main chain (Table 1). Branched XOS products often have two unsubstituted xylose residues at the reducing end and the side groups at the non-reducing end (Mathew et al. 2018). While their ability to act on insoluble xylan is rather low, substitutions at the xylose backbone do not interfere greatly. Due to the low sequence conservation in the aglycone subsites, different hydrolysis products are generated by different members of the family (Pell et al. 2004; Linares-Pastén et al. 2018).

**Table 1:** Substituents allowed in subsites of xylanases (Based on Linares-Pastén et al. 2018)

Family	Substituent	Glycone subsites			Aglycone subsites		
		− 3	− 2	− 1	+ 1	+ 2	+ 3
GH5_34	Araf		P	N	P	P	
GH10	Araf		P	B	P/B	P/B	P/B
	MeGlcA	P	B	B	P/B		
GH11	Araf	P	P/B	B	B	P	
	MeGlcA	P/B	P/B	B	B	P	
GH30_8^a^	MeGlcA		N	B			

*P* permitted, *B* banned, *N* necessary, *P/B* not conserved, permitted in some, banned in others

^a^Only typical GH30_8 xylanases with glucuronoxylanase activity are considered

All enzymes belonging to the GH11 family are xylanases with a conserved structure exhibiting the β-jelly roll fold characteristic for clan C enzymes (Fig. 2b). Their active sites can comprise up to seven subsites; however, five (− 2 to + 3) are most common. Three consecutive unsubstituted xylose monomers are required for attack of the xylan main chain by GH11 xylanases. The enzymes cleave the main chain on glycosidic linkage prior to the xylose monomer carrying a substitution and leave the following two linkages unaffected. Therefore, branched XOS products generated by GH11 xylanases typically have two unsubstituted xylose monomers at the reducing end (Mathew et al. 2018). Side groups at the xylan backbone are generally less tolerated than in GH10 enzymes due to the narrow binding cleft with no acceptance in the − 1 and + 1 subsites (Biely et al. 1997; Paes et al. 2012; Wan et al. 2014).

The production of specific substituted XOS cleavage products, including AXOS and uronic acid xylooligosaccharides (UXOSs), has successfully been performed with members of both GH10 and GH11 families (Falck et al. 2014; Tian et al. 2015). However, xylanases belonging to family 10 are preferred due to their open substrate-binding cleft which can, for example, take in arabinose side groups at the − 2 and + 1 subsites and 4-O-methyl-d-glucuronic acid side groups at the − 3 and + 1 subsites (Mathew et al. 2017) (Table 1). However, if the production of AXOS or UXOS with a larger DP from low substituted xylan is desired, the use of GH11 enzymes might be more relevant. Those can accommodate arabinose side groups at the − 3, − 2, and + 2 subsites and 4-O-methyl-d-glucuronic acid side groups at the − 3 and + 2 subsites (Table 1) (Linares-Pastén et al. 2018; Pollet et al. 2010). Insoluble arabinoxylan (AX) is more efficiently cleaved by members of the GH11 family due to their smaller size (< 30 kDa), which enables them to penetrate the cell wall matrix faster (Falck et al. 2014; Linares-Pastén et al. 2018). In order for GH10 xylanases to act equally efficiently on insoluble AX, a pretreatment step is required (Falck et al. 2014).

The specific activity of commercially available endoxylanases varies within a wide range and is of course dependent on the substrate type and reaction conditions. The activity on wheat arabinoxylan is generally somewhat higher for the GH11 enzymes than for the GH10 enzymes, with 1500 U/mg for the GH11 enzyme from *Neocallimastix patriciarum* as the most efficient one of those available from Megazyme. This high specific activity indicates a potential for industrial applications.

## GH5 arabinoxylanases

Glycoside hydrolase family 5 includes enzymes active on a large variety of β-linked substrates. Concerning production of substituted XOS, subfamily 34 is of special interest, since it involves xylanases requiring arabinose substitution, therefore called arabinoxylanases (EC. 3.2.1.-). The first characterized enzyme in GH5_34 is the one from *Clostridium thermocellum* (*Ct*Xyl5A) (Correia et al. 2011). *Ct*Xyl5A has no activity on birch and beech xylan or on glucuronoxylan. However, it is highly active on wheat and rye arabinoxylan, although the K_m_ values are high. When *Ct*Xyl5A is hydrolyzed rye AX, it formed a variety of AXOS but no unsubstituted XOS (Falck et al. 2018). Debranching of the hydrolysis products with weak acid produced xylose and XOS with DP2–10. More detailed analysis of AXOS produced from the hydrolysis of rye AX showed that they all (> 99%) have an α-1-Araf side chain on O3 of the reducing end Xylp residue, and some of them had additional α-1-Araf side chains (Correia et al. 2011).

Structural studies have shown that the xylan binding site of *Ct*Xyl5A has an open cleft, thereby allowing for arabinose substitution in all subsites − 2 to + 2 (Correia et al. 2011) (Fig. 2c). Interestingly, arabinose substitution in subsite − 1 is required (Table 1) or else the substrate will not be hydrolyzed. The critical side chain arabinose binds in a separate subsite − 2*, which has conserved Glu, Tyr, and Asn residues. Computational studies have shown that the substrate is strongly bound only in the − 1 and − 2* subsites (Falck et al. 2018).

*Ct*Xyl5A is a multidomain protein. In addition to its catalytic module, it contains three carbohydrate binding modules (CBM6, CBM13, and CBM62): one fibronectin type 3 domain and two dockerin domains (Labourel et al. 2016). The dockerin domains can connect the enzyme to the cellulosomes on the cell surface and are thus assumed to take part in the plant cell wall degrading machinery of the organism. The CBMs stabilize the enzymes and CBM62 binds to d-galactopyranose and l-arabinopyranose. This indicates that the enzyme might be involved in the breakdown of complex xylans containing d-galactose in their side chains.

In addition to *Ct*Xyl5A, three additional GH5_34 enzymes have been studied and they are all active on arabinose-substituted xylans but not Birchwood xylan (Labourel et al. 2016). The ones originating from *Acetivibrio cellulolyticus* (*Ac*GH5) and *Verrucomicrobiae bacterium* (*Vb*GH5) are multidomain proteins having 2–3 CBMs and in the case of *Vb*GH5, an additional catalytic GH module (GH43_10), whereas *Ac*GH5 has a catalytic carbohydrate esterase module. On the other hand, the enzyme from the fungus *Gonapodya prolifera* (*Gp*GH5) just contains the catalytic module, indicating that it targets simpler substrates than the other enzymes from the subfamily. When acting on arabinoxylans from wheat, rye, and corn, *Vb*GH5 produced much less low-molecular-weight products than the other enzymes, indicating that it is more restricted concerning cleavage sites in AX substrates.

The specificity of GH5_34 xylanases makes them useful for the degradation of highly arabinose substituted xylans, which are resistant to degradation by GH10 and GH11 xylanases. Furthermore, these enzymes are promising tools for the preparation of AXOS, without significant formation of unsubstituted XOS.

## GH30 glucuronoarabinoxylan endoxylanases

Currently, all enzymes termed glucuronoarabinoxylan endo-β-1,4-xylanases (EC3.2.1.136) are classified under GH30, but are like GH 5 and 10, members of clan A, sharing the TIM-barrel (α/β)_8_ fold (Linares-Pastén et al. 2018). The first isolated candidates were initially members of GH5 but were reclassified as phylogenetic analysis showed higher similarity to GH30 (St John et al. 2010). To date (6 June, 2018), subfamily 8 in GH30 (GH30_8) holds 16 characterized candidates (15 bacterial and 1 eukaryotic), and 6 bacterial enzymes have been structure determined (www.cazy.org). They generally differ from the endoxylanases in family 10 by having high selectivity for glucuronoxylan and XOS substituted with glucuronic acid (GlcA) or methylglucuronic acid (MeGlcA) via an α-1,2 linkage. This is combined with lower selectivity for unsubstituted xylan, AX, and XOS, which may be structurally reflected by more open-binding clefts (Sainz-Polo et al. 2014). (At least one exception from this rule is however known as the enzyme from *C. papyrosolvens* (St John et al. 2014) is active on non-substituted xylan).

Typical for the overall fold of GH30 enzymes is the 9-stranded β-sandwich structure that connects to the N- and C-terminal of the TIM-barrel catalytic module (Fig. 2d). The arrangement of this structure divides GH30 into 9 subfamilies, where the glucuronoarabinoxylan endo-β-1,4-xylanases are classified under subfamily 8 (GH30_8). In some cases, the β-structure is shown to be involved in binding of glucuronoxylan, but this is not a general feature. Other enzymes are shown to be connected to a CBM, e.g. CBM35 of Xyn30D from *Paenibacillus barcinonensis* (Sainz-Polo et al. 2014) that is reported to be glucuronoxylan binding. Overall, GH30_8 candidates differ in modularity, some enzymes being single module, while others are connected to binding modules or parts of cellulosomes.

The active site in GH30_8 has been reported to hold five subsites, with the three aglycone subsites (− 3, − 2, − 1) being most conserved and with strong interactions to the substituent in subsite − 2 (Fig. 2d) shown for the enzymes from *Bacillus subtilis* (St John et al. 2011) and *Erwinia chrysanthemi* xylanase A (Urbanikova et al. 2011) (Table 1). Suchova et al. (Suchova et al. 2018) also recently published a study on the importance of interaction with residue Arg293 and the glucuronic acid for the specific cleavage in the *E. chrysanthemi* enzyme.

Despite the increasing structural knowledge on GH30_8, there are thus far relatively few reports focusing on production of acidic (glucuronosylated) xylooligosaccharides (UXOSs). Rhee et al. (2014) and Wei et al. (2016) have published studies on UXOS produced from sweet gum wood and sorghum, respectively, using a combination of enzymes from *B. subtilis*, including GH30. A recent study also shows the potential of UXOS as oligosaccharides with antioxidant activity, where UXOS produced by Xyn30D from *P. barcinonensis* form birch and beechwood showed higher antioxidizing activity and produced longer oligosaccharides than XOS produced from the same material using a GH10 enzyme (Valls et al. 2018).

## How are XOS, AXOS, and UXOS metabolized in the gut?

The microbiota of the human gastrointestinal (GI) tract is a complex system consisting of more than 2000 identified species, classified into 12 different phyla, of which more than 90% belong to Proteobacteria, Firmicutes, Actinobacteria, and Bacteroidetes (Flint et al. 2012; Thursby and Juge 2017). Food components which are not degraded by the human enzymes can be fermented by the gut microbiota. XOS and AXOS have been more studied than UXOS and shown to selectively stimulate the growth of probiotic gut bacteria conferring positive effects to the host health (Broekaert et al. 2011). The most well-known probiotic strains belong to the *Bifidobacterium* (Actinobacteria) and *Lactobacillus* (Firmicutes) genera. These bacteria have quite different (A)XOS utilization systems. *Bifidobacterium adolescentis* has been shown to consume AXOS and undecorated XOS, while *Lactobacillus brevis* utilized only XOS (Falck et al. 2013). Likewise, *Weissella confusa/cibaria* (Firmicutes), which are putative probiotics, showed ability to use XOS but not AXOS (Patel et al. 2013). Transcriptomics and structural studies on *B. animalis* support a mechanism of XOS and AXOS uptake based on their capture and transport through ABC transporters (Ejby et al. 2013). The depolymerization of XOS and AXOS then takes place inside the cell where the arabinosyl and acetyl substituents are removed by GH43 arabinofuranosidases and acetyl esterases respectively, while the main chain is degraded by GH43 xylosidases.

In the gut microbiota, cross feeding is an important mechanism for the degradation of complex substrates. Strains producing arabinofuranosidases can thus utilize the arabinose substituents of AXOS, while other strains can consume the unsubstituted XOS formed. Likewise, *Bacteroides* strains producing α-glucuronidases have been shown to remove glucuronic acid substituents leaving XOS for *Bifidobacteria* and other bacteria (Ohbuchi et al. 2009).

The most important effects of xylooligosaccharides in the human diet are as follows: (1) they modulate the composition of the gut microbiota; (2) their fermentation produces beneficial metabolites, such as short chain fatty acids; and (3) they can be designed to be fermented with different rates, thereby inducing beneficial fermentation in the whole colon.

### Modulation of the gut microbiota

Relatively many intestinal bacteria can grow on XOS (Crittenden et al. 2002). However, human studies have shown that consumption of XOS products causes an increase in fecal *Bifidobacteria* (Lecerf et al. 2012), which indicates that these are selectively promoted by the products and most probably are responsible for a substantial part of their degradation. Since AXOS and UXOS can be utilized by fewer strains than XOS, they are more selective in their modulation of the gut microbiota. It has been demonstrated that UXOS can be utilized by only few of the human fecal *Bifidobacteria*, while many of them grew on XOS (Ohbuchi et al. 2009). Furthermore, using human gut models, it was shown that long-chain AX promoted the growth of *B. longum* strains, while the established prebiotic inulin stimulated other *Bifidobacteria* (Van den Abbeele et al. 2013).

### Fermentation products

XOS with different substitutions fermented by the gut microbiota produce mainly short-chain fatty acids (acetate, propionate, and butyrate), lactate, CO_2_, and H_2_. Relative amounts of these products vary depending of the type of substituent(s) in the oligosaccharide. In vitro fermentations using human fecal inocula have shown production of mainly acetate and lactate when linear XOS and AXOS of low molecular weight (DP 2–11) were used (Kabel et al. 2002). The same study showed increased production of propionate and butyrate and lower lactate when acetyl and 4-O-methyl-glucuronyl groups were the substituents (i.e. UXOS).

### Fermentation rate

Rapid fermentation in the colon can produce discomfort and poor tolerability, and thus it is beneficial if at least a part of the fibers in the diet is fermented slowly. It was suggested (Pollet et al. 2012) that the lower fermentability of complex AXOS could make them persist longer in the colon, and their fermentation takes place partially in the distal parts of the colon, suppressing the protein fermentation. Protein fermentation in the colon produces potentially toxic compounds, such as ammonia, phenols, and thiols, and therefore, its reduction is desirable.

Treatment of arabinoxylan extracted from Brewer’s spent grain with a GH11 xylanase did not speed up its fermentation by fecal bacteria, thus indicating that cleavage of the main xylan chain was not a rate-limiting step (Sajib et al. 2018). It has been shown that linear XOS and AXOS are fermented more rapidly than oligosaccharides containing acetyl groups and glucuronic acid (Kabel et al. 2002). Other indications of the importance of substituents were found in a recent study of structural features of soluble cereal fibers associated with a slow fermentation by human fecal microbiota in vitro. Such features included terminal xylose in branches and arabinose containing trisaccharides as substituents on the main chain (Rumpagaporn et al. 2015).

## Concluding remarks

In order to create ideal prebiotic products, there is a need for research on the following topics:

- Which is the ideal composition of the gut microbiota?
- Which prebiotic products can induce the ideal gut microbiota?
- How can these prebiotic products be produced?

There are still knowledge gaps in these fields, but significant progress is being made. Xylans seem to be very attractive raw materials to produce prebiotic products, and endoxylanases are very useful tools to tailor the xylans to become effective prebiotics. The rapid progress in the field of carbohydrate active enzymes makes it likely that many xylanases with novel specificities will be discovered in the coming years, which will further increase the scientific importance of this group of enzymes. Finding native enzymes with high enough catalytic activity or developing enzymes to achieve this will be important for industrial applications.
